# The effects of trauma on feedback processing: an MEG study

**DOI:** 10.3389/fnins.2023.1172549

**Published:** 2023-11-02

**Authors:** Abdulrahman S. Sawalma, Christian M. Kiefer, Frank Boers, N. Jon Shah, Nibal Khudeish, Irene Neuner, Mohammad M. Herzallah, Jürgen Dammers

**Affiliations:** ^1^Institute of Neuroscience and Medicine (INM-4), Forschungszentrum Jülich GmbH, Jülich, Germany; ^2^Faculty of Medicine, RWTH Aachen University, Aachen, Germany; ^3^Palestinian Neuroscience Initiative, Al-Quds University, Abu Dis, Palestine; ^4^Faculty of Mathematics, Computer Science and Natural Sciences, RWTH Aachen University, Aachen, Germany; ^5^Institute of Neuroscience and Medicine (INM-11), Jülich Aachen Research Alliance (JARA), Forschungszentrum Jülich GmbH, Jülich, Germany; ^6^Jülich Aachen Research Alliance (JARA)-Brain – Translational Medicine, Aachen, Germany; ^7^Department of Neurology, University Hospital RWTH Aachen, Aachen, Germany; ^8^Department of Psychiatry, Psychotherapy and Psychosomatics, RWTH Aachen University, Aachen, Germany; ^9^Center for Molecular and Behavioral Neuroscience, Rutgers University, Newark, NJ, United States

**Keywords:** trauma, PTSD, magnetoencephalography, feedback-based learning, spatio-temporal cluster permutation test

## Abstract

The cognitive impact of psychological trauma can manifest as a range of post-traumatic stress symptoms that are often attributed to impairments in learning from positive and negative outcomes, aka reinforcement learning. Research on the impact of trauma on reinforcement learning has mainly been inconclusive. This study aimed to circumscribe the impact of psychological trauma on reinforcement learning in the context of neural response in time and frequency domains. Two groups of participants were tested - those who had experienced psychological trauma and a control group who had not - while they performed a probabilistic classification task that dissociates learning from positive and negative feedback during a magnetoencephalography (MEG) examination. While the exposure to trauma did not exhibit any effects on learning accuracy or response time for positive or negative feedback, MEG cortical activity was modulated in response to positive feedback. In particular, the medial and lateral orbitofrontal cortices (mOFC and lOFC) exhibited increased activity, while the insular and supramarginal cortices showed decreased activity during positive feedback presentation. Furthermore, when receiving negative feedback, the trauma group displayed higher activity in the medial portion of the superior frontal cortex. The timing of these activity changes occurred between 160 and 600 ms post feedback presentation. Analysis of the time-frequency domain revealed heightened activity in theta and alpha frequency bands (4–10 Hz) in the lOFC in the trauma group. Moreover, dividing the two groups according to their learning performance, the activity for the non-learner subgroup was found to be lower in lOFC and higher in the supramarginal cortex. These differences were found in the trauma group only. The results highlight the localization and neural dynamics of feedback processing that could be affected by exposure to psychological trauma. This approach and associated findings provide a novel framework for understanding the cognitive correlates of psychological trauma in relation to neural dynamics in the space, time, and frequency domains. Subsequent work will focus on the stratification of cognitive and neural correlates as a function of various symptoms of psychological trauma. Clinically, the study findings and approach open the possibility for neuromodulation interventions that synchronize cognitive and psychological constructs for individualized treatment.

## 1. Introduction

According to the Diagnostic and Statistical Manual, 5th edition, (DSM-5), exposure to psychological trauma can induce four clusters of symptoms: intrusive re-experiencing of events related to the traumatic event, persistent avoidance of stimuli or memories associated with the traumatic event, negative alteration in cognition and mood and symptoms of arousal (American Psychiatric Association, 2013). For a diagnosis of post-traumatic stress disorder (PTSD) to be made, the severity and frequency of these post-traumatic stress symptoms (PTSS) must reach a threshold for diagnosis (Megías et al., 2007). However, it is also recognized that subthreshold PTSS are debilitating in their own right (Schnurr et al., 2000; Brancu et al., 2016; Kim et al., 2020). Compared to individuals without trauma exposure, people with PTSS may experience social and functional impairments, suicidal ideations, and other psychiatric problems, including anxiety and depression (Marshall et al., 2001; Zlotnick et al., 2002; Friedman et al., 2011; Morgan-López et al., 2020).

Classically, PTSS were attributed to impairments in Pavlovian conditioning, extinction, or recall of fear (Lissek and van Meurs, 2015). However, limited research has examined reinforcement learning and the associated neural dynamics following exposure to trauma. Traumatic experiences are known to reduce positive feedback expectancy and satisfaction (Hopper et al., 2008), decrease the ability of the person to exploit information about positive feedback in their environment (Hanson et al., 2017) and increase their sensitivity to negative stimuli (Sawyer et al., 2016). However, little is known about the dynamics of brain signals during feedback processing following traumatic experiences and how it relates to learning from positive and negative feedback.

Exposure to trauma has been shown to affect the neural circuitry for feedback processing, including multiple cortical and subcortical structures. Indeed, exposure to trauma has been associated with decreased activation of the medial prefrontal cortex (mPFC; encodes stimulus value; Sescousse et al., 2013; Purves et al., 2018), higher activation in the amygdala (important for fear conditioning; Greco and Liberzon, 2016), lower connectivity between the anterior cingulate cortex (ACC; assessing feedback based on choices; Purves et al., 2018) and hippocampus (memory formation; Alvarez and Squire, 1994), and increased connectivity between the insula (integration of internal and somatic states; Craig, 2002; Sescousse et al., 2013; Purves et al., 2018) and other regions in the salience network (Sripada et al., 2012). Furthermore, trauma exposure has also been associated with a decrease in the size of the amygdala, insula, ACC, and mPFC (Ganzel et al., 2008). However, although response to positive and negative feedback can explain some trauma-related symptoms, it is still unclear how trauma exposure affects online processing of feedback with temporal and spatial precision.

Studying the temporal and spectral aspects of feedback processing can provide a better understanding of the cognitive effects of trauma exposure. Given that feedback components follow reproducible patterns of activity that are time- and frequency-dependent (Bernat et al., 2015), studying these components can shed more light into the processes underlying them. Previous studies have relied on electroencephalography (EEG) to investigate the effect of trauma on the temporal dynamics of feedback processing following trauma (Pechtel and Pizzagalli, 2013; Lieberman et al., 2017). However, while EEG provides a high temporal resolution, when compared to other modalities, such as fMRI, it has very low spatial resolution (Glover, 2011). To bridge the gap between temporal and spatial resolution, magnetoencephalography (MEG) would be the ideal resort (Hämäläinen et al., 1993). To date, only a handful of studies have used MEG to characterize feedback signal components in healthy individuals and compared them to EEG literature (Miltner et al., 2003; Keil et al., 2010; Talmi et al., 2012). MEG studies addressing psychological trauma have mainly focused on studying resting state (Huang et al., 2014; James et al., 2021, 2022), face-processing tasks (Badura-Brack et al., 2018) or working-memory tasks (McDermott et al., 2016). However, to the best of the authors’ knowledge, no MEG study has investigated feedback processing in the context of psychological trauma.

We hypothesize that traumatic experiences affect feedback processing, which will be reflected as differences in activity of the involved regions in the temporal and frequency domains. To test this, we examine the spatio-temporal and spectro-temporal facets of feedback processing in the context of trauma exposure. A feedback-based learning task was administered to two groups of individuals - a group with a history of trauma exposure and a control group without such history - during an MEG examination. As a difference in the cortical brain regions involved in the processing of positive and negative feedback was expected between the two groups, feedback processing differences in the temporal and spectral domains were further investigated. This is the first study to examine the potential changes in spatial, spectral, and temporal aspects of feedback processing following traumatic events.

## 2. Methods

### 2.1. Experimental paradigm

**Figure 1:**
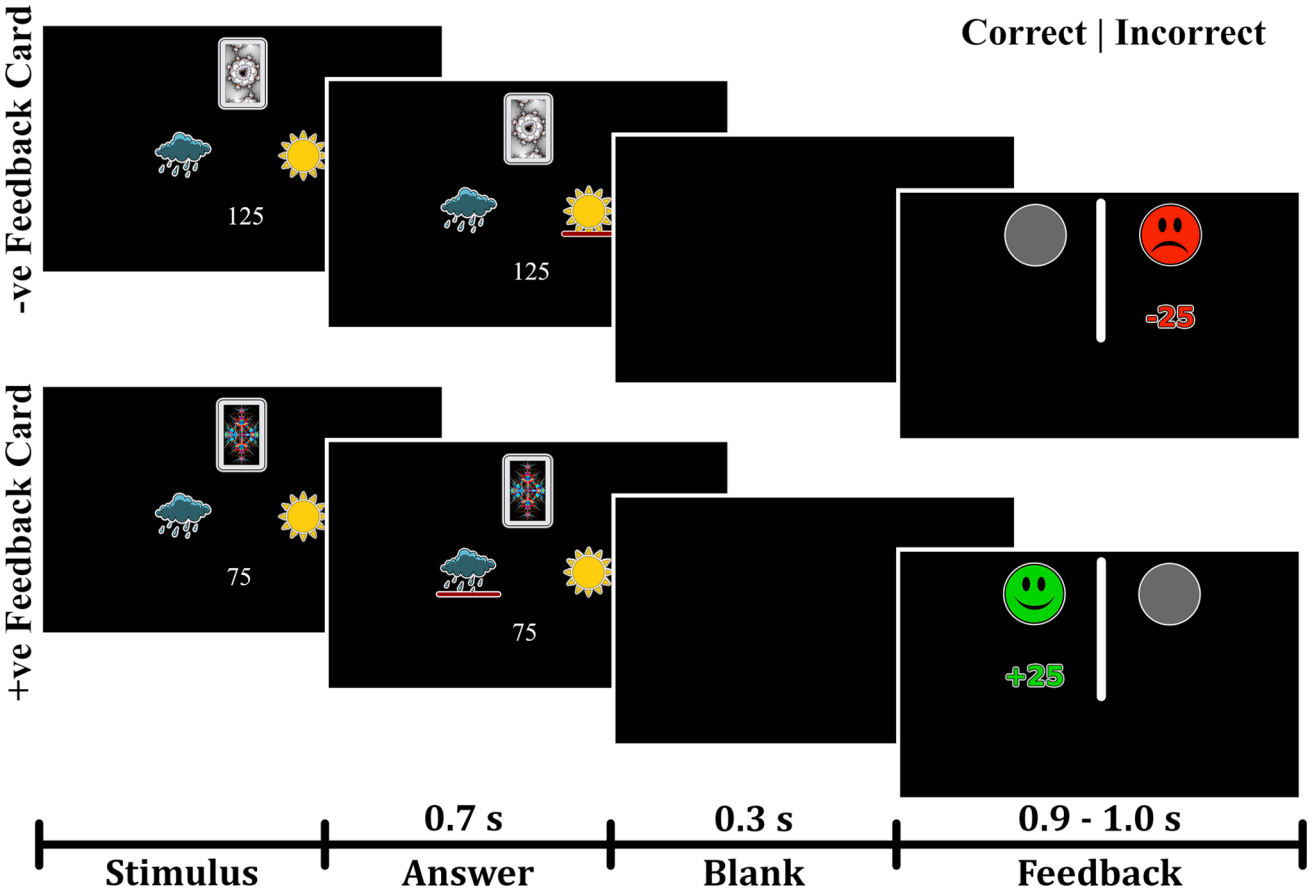
Experimental paradigm of the probabilistic task. The task paradigm for negative-feedback stimuli is illustrated in the upper portion. Participants received negative feedback when they provided an incorrect answer and no feedback when they gave a correct answer. The lower portion depicts the paradigm for positive-feedback stimuli, where participants received positive feedback for correct answers and no feedback for incorrect answers.

Participants were administered a probabilistic classification task that dissociates learning from positive and negative feedback (Herzallah et al., 2017). On each trial, one of four fractal images was presented to the participant, and they were asked to guess whether a fractal image (stimulus) predicts weather as “sun” or “rain.” The stimulus remained visible until the participant responded. A red line below the “sun” or “rain” image indicated the chosen answer for 700 milliseconds, followed by a 300 ms blank screen (Figure 1). Feedback was presented for a variable time of between 900 and 1,000 ms as either a green smiley face with the text “+25,” representing positive feedback; a red frowny face with the text “-25,” representing negative feedback; or a gray circle without text, representing no feedback. Two of the stimuli had a 90% probability of predicting “sun” and a 10% chance of predicting “rain.” The other two stimuli predicted “rain” with a 90% probability and “sun” with a 10% probability. For feedback type, two stimuli (positive-feedback stimuli) were associated with positive feedback when answered optimally and no feedback when answered non-optimally. The other two stimuli (negative-feedback stimuli) were associated with negative when answered non-optimally and no feedback when answered optimally. Task structure of the task is shown in Table 1.

**Table 1:** Structure of the probabilistic classification task.

Stimulus	Feedback type	Card type
S1	If correct: +25 If incorrect: 0	Random assignment per participant: 2 cards: “sun” 2 cards: “rain”
S2		
S3	If correct: 0 If incorrect: −25	
S4		

Two stimuli give positive or no feedback, and the other two give negative or no feedback. The assignment of “sun” and “rain” to the four stimuli is done at random and is fixed per participant.

The task consisted of three blocks, each containing 160 trials, and the participants needed an average duration of 24.7 ± 3.8 min to finish the task. Stimulus presentation was performed using the Python package PsychoPy 2.0 (Peirce et al., 2019). The visual stimuli were presented on an MEG-compatible screen using a Barco FL35 WUXGA projector with a resolution of 1980 × 1,200 pixels and a frequency of 60 Hz.

### 2.2. Participants

Seventy-nine participants between the ages of 21 and 43 were recruited using flyers, social media, and snowball sampling. All participants were interviewed by a trained researcher who administered the mini-international neuropsychiatric interview (MINI; Sheehan et al., 1998). Using the MINI, the presence of trauma was determined, and participants were divided into two groups, the trauma-exposed group (Trauma) and the no-trauma-exposed control group (Control). The study was conducted according to the criteria of the Declaration of Helsinki and was approved by the ethics committee of RWTH Aachen University Hospital, Germany. Following an explanation of the procedure, written informed consent was collected from every participant at the beginning of the session.

For inclusion in the Trauma group, participants were required to have had a traumatic event (criterion A) as defined by the MINI. The rest were assigned to the Control group. Using the MINI, comorbid psychiatric conditions were determined. The presence of psychiatric disorders, as defined by the MINI, was only accepted in the Trauma group. From the sample taken, 14 participants in the Trauma group met the criteria of either major depressive disorder, dysthymia, suicidality, social phobia, obsessive-compulsive disorder, or generalized anxiety disorder. The inclusion criteria for the Control group constituted the absence of any traumatic event and the absence of any psychiatric disorders at the time of testing, which was confirmed using the MINI. The exclusion criteria for all groups were the current use of psychotropic drugs, left-handedness, inability to understand the computer-based task, and major neurological or medical illnesses, including endocrine disorders. After the exclusion of three participants, the sample included 76 participants: 56 in the Trauma group and 20 in the Control group. Participant demographics are presented in Table 2.

**Table 2:** Demographics of the tested sample.

	Control	Trauma
Count	20	56
Female Percentage	15.0	23.2
Age (mean ± SD)	29.0 ± 5.0	30.7 ± 7.6
Education (mean ± SD)	18.6 ± 2.0	15.9 ± 4.4

Age and education are shown as mean ± standard deviation (SD).

To improve data accuracy and reduce noise, any trials with high signal amplitudes (above 5 picotesla) were excluded from the MEG analysis. As a result, one participant was excluded from the positive-feedback analysis, and two participants were excluded from the negative-feedback analysis. The final number of participants included in each of the analyses is shown in Table 3.

**Table 3:** Number of participants included in each of the analyses.

Type of Analysis	Control	Trauma
Behavioral analysis	20	56
MEG analysis – positive-feedback trials	20	55
MEG analysis – negative-feedback trials	20	54

### 2.3. Behavioral analysis

In order to investigate whether trauma affects feedback processing and perception, learning accuracy and reaction time was analyzed for both positive and negative feedback. In order to avoid the multiple comparison problem, a one-way Multivariate analysis of variance (MANOVA) was initially performed with positive feedback accuracy, negative feedback accuracy, positive feedback reaction time, and negative feedback reaction time as dependent variables; and Group as the independent variable.

Due to time constraints for some participants and the tight schedule for testing, not all participants completed all 480 trials. Sixty-nine participants completed all 480 trials, one participant completed 416 trials, five participants completed 320 trials, and one participant only completed 160 trials. To compensate for the difference in the number of trials completed, behavioral analysis was performed once on the full data set for each participant and once on the first 160 trials only.

In order to obtain greater insight into the effect of trauma on feedback-based learning, the percentages of “learners” and “non-learners” were calculated for both positive- and negative-feedback trials. “Learners” of a particular feedback were defined as those who had more than 65% correct responses (Myers et al., 2013) for either of the feedback cards. The percentage of learners between the two groups was compared using the chi-square test.

### 2.4. MEG data acquisition

MEG data were collected using the Magnes-3600WH MEG system from 4d-Neuroimaging (San Diego, United States of America). Brain activity was measured with a sampling rate of 1017.25 Hz using 248 magnetometers. Additionally, cardiac and ocular activity were collected using the BrainAmp ExG MR amplifier (Brain Products, Gilching, Germany) with a sampling frequency of 5,000 Hz. This signal was down-sampled to 1017.25 Hz and merged with the MEG signal into a single recording. Electrooculography (EOG) signals were collected by attaching two electrodes lateral to the eyes and two electrodes above and below one of the eyes to record eye movements and eye blinks, respectively. Electrocarduigraphy (ECG) signals were collected by attaching an electrode at the middle of the right clavicle, one electrode at the left side below the apex of the heart, and a third electrode at the left leg. Finally, three head location coils were attached to the head to measure its relative position in space before and after each experiment.

### 2.5. MEG-MRI co-registration

To obtain anatomical information about the head and brain, MR measurements were taken from all participants at either a 3 T PRISMA scanner (Siemens, Erlangen, Germany) using the MPRAGE sequence (Mugler and Brookeman, 1990) or at a 7 T MAGNETOM Terra scanner (Siemens Healthineers, Erlangen, Germany) using the MP2RAGE sequence. As the participants of the Trauma group were also included in another study that used the 7 T MR system, images obtained at 7 T were used for this group to avoid further inconvenience. A sanity check was manually performed for each MEG file after the co-registration step to ensure that the use of the two MR systems did not affect the source localization of the signal. This was conducted by recording the mean error of co-registration and ensuring it was less than 3 mm.

MEG brain activity was aligned with the structural information by means of source (i.e., brain) space construction utilizing the FreeSurfer package (Dale et al., 1999; Fischl et al., 1999). For source localization, dynamic statistical parametric mapping (dSPM) was applied with a depth weighting of 0.8 (Dale et al., 2000). The individual source estimates were then projected onto the average template brain, as provided by FreeSurfer, using 8,196 vertices with an average distance between vertices of about 5 mm. Finally, the source activity was divided into anatomical regions based on the areas defined by the Desikan-Killiany atlas (Desikan et al., 2006). Co-registration from the MEG to the MRI coordinate space and the solving of the forward and inverse problems was performed using the MNE-Python library (Gramfort et al., 2014).

Three participants did not have an MR scan or had a corrupt file, so the average MR brain shape provided by Freesurfer (fsaverage template brain) was used instead. To ensure the quality of co-registration for all participants, including the three participants with missing files, the mean error of co-registration was recorded to be less than 3 mm using mne-coreg interface (Gramfort et al., 2013).

### 2.6. Preprocessing the MEG signal

Strong artifacts in MEG channels were identified using an in-house algorithm based on density-based spatial clustering of applications with noise (DBSCAN), as implemented in scikit- learn (Ester et al., 1996; Pedregosa et al., 2011). This was followed by visual inspection to identify noisy channels missed by this function. The signal of the identified “bad” channels was replaced by an interpolated signal from surrounding channels (Perrin et al., 1989). Environmental noise and powerline noise was removed by subtracting individually weighted reference channels from each of the MEG channels (Robinson, 1989).

Biological noise (i.e., ocular and cardiac signals) was removed using independent component analysis (ICA; Hyvärinen and Oja, 2000). The signal was split into segments with an average of 155.1 s per segment, then bandpass filtered (1–45 Hz) to improve the quality of the ICA decomposition (Winkler et al., 2015). Ocular activity was detected by finding components with a Pearson’s correlation of 0.3 or more with the EOG channel, while cardiac activity was detected using cross-trial phase statistics (CTPS; Dammers et al., 2008). A visual inspection of the results was finally performed to ensure all EOG and ECG signals were removed. The components were then projected back to the data. The average number of removed components was found to be 7.0 for all participants. Dividing the groups into Trauma and Control, the average number of removed components for the Trauma group is 7.1, while the average for the Control group is 6.5, with no significant difference between the two groups (*p*-value = 0.453).

### 2.7. Creating epochs

In order to analyze the data in the spatial, temporal and spectral domains, epochs were extracted around the event of interest (i.e., positive or negative feedback) starting 250 ms prior to the event and extending to 600 ms after the event, with the time 0 representing the feedback onset. The interval − 250 – 0 ms was chosen as a baseline. This means that, for each of the analyses, the mean of the signal in the baseline interval was subtracted from the entire signal of the epoch. The signal was then divided by the standard deviation of the baseline signal.

**Figure 2:**
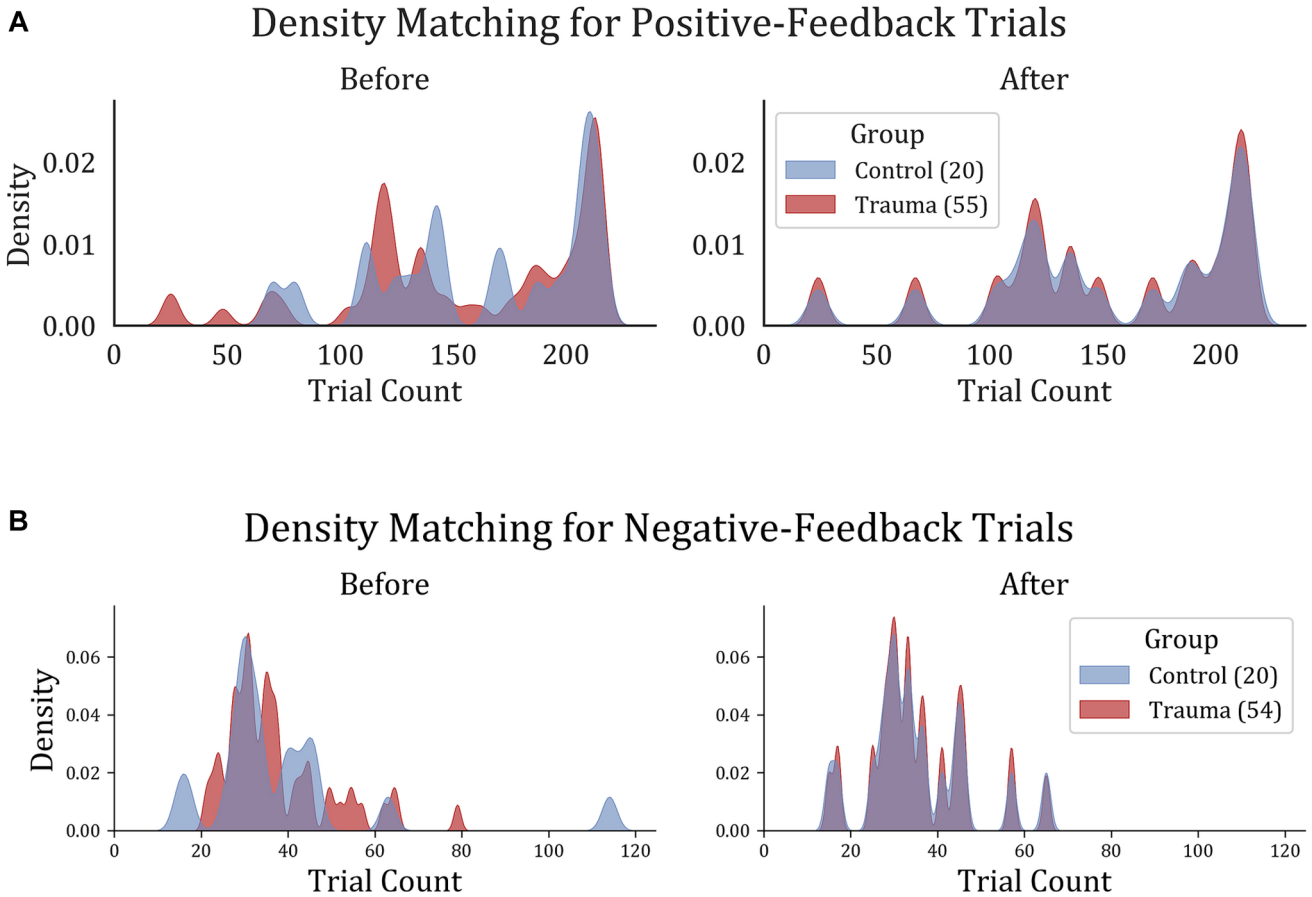
Trial density matching for **(A)** positive-feedback and **(B)** negative-feedback trials. Trauma and Control groups were matched in trials in order to remove the bias that might result from the difference in trial count per group.

To ensure comparable signal-to-noise ratios between the two groups, the number of trials was equalized between the two groups by removing trials from the participants with larger trial counts until the density distribution of trials was equal. This was done by dividing the data into 20 bins, each containing one participant from the Control group and 2–3 participants from the Trauma group. Within each bin, the number of trials was equalized to match the participant with the lowest number of trials. For positive-feedback trials, the average number of trials became similar to those in the trauma group (Control: min = 24, max = 215, mean = 155; Trauma: min = 24, max = 215, mean = 154). The case was also similar for negative-feedback trials (Control: min = 15, max = 65 mean = 35 for both groups). The results of the density matching are illustrated in Figure 2.

### 2.8. Regions of interest

Regions of interest were defined as regions that showed significantly-different activity in space and time between the two groups (Control vs. Trauma) in positive and negative-feedback trials separately. The Monte-Carlo-based non-parametric spatio-temporal cluster permutation test (SCPT) was performed to extract differences between the two groups (Maris and Oostenveld, 2007). Since SCPT only performs a single statistical test on the entire data set, the multiple comparison problem does not apply. This means that differences across conditions directly reflect the significance level (Maris and Oostenveld, 2007).

SCPT was performed using a two-sample permutation t-test with 10^4^ permutations. In order to choose the cluster threshold, the difference in trial numbers between the two conditions was taken into account. As the number of trials was much smaller for negative-feedback trials (35 vs. 154), the significant clusters were likely to have a shorter duration of activity. Therefore, the cluster threshold for positive-feedback trials was chosen to be equivalent to a *t*-value at an alpha level of 0.05 (corresponding to a threshold value of 1.99), while for negative-feedback trials, it was chosen to be equivalent to a *t*-value at an alpha level of 0.001 (corresponding to a threshold value of 3.43). The choice of cluster threshold does not affect the false alarm rate, but it does affect the sensitivity of the test (Maris and Oostenveld, 2007). Finally, the significance threshold for both types of comparisons was set to 0.05.

To ensure that the resulting areas did not include outliers in space or time, clusters with 15 vertices or less in the template brain (corresponding to an active cortical area of about 2.29 cm^2^) were excluded. Similarly, temporally short clusters with a duration of less than 20 ms were discarded from the analysis. Finally, vertices within the medial wall of the brain were also excluded from the analysis. As previously stated, this test was conducted for both positive-feedback and negative-feedback trials separately. The resulting regions of interest were used to further conduct the time-course analysis and the spectro-temporal analysis.

### 2.9. Time-course analysis

In order to investigate possible changes in the temporal dynamics of feedback processing after trauma, positive feedback-related activity for Control vs. Trauma was compared on positive-feedback trials. For this comparison, a representative source time course (rSTC) was computed for each of the ROIs identified in the previous step using the mean activation of all “significant” vertices within the respective ROI. These representative time courses represent the average of epochs and vertices for a given ROI. Afterwards, all rSTCs were z-scored using the mean and standard deviation from the pre-stimulus interval.

To identify differences in the temporal dynamics between the groups, a cluster permutation test was applied in a similar way to the SCPT described above, but only in the temporal domain, so as to investigate differences between the ROI activation time courses (Maris and Oostenveld, 2007). Cluster permutation tests were applied using a paired *t*-test with a clustering threshold using a critical alpha level of 0.05, which corresponds to a *t*-threshold of 1.99, and a significance level of *p* < 0.05. The number of permutations used was set to 10^4^ permutations.

To study how the activity in brain regions affect learning, the two groups are divided into “learners,” with more than 65% accuracy, and “non-learners.” The signal within the significant time intervals (time intervals of interest; TOIs) of each rSTC was extracted for each subject to be compared between the two groups. This was conducted separately for Trauma and Control groups to control for trauma-related effects.

### 2.10. Spectro-temporal analysis

In addition to the statistical analysis in the temporal domain, differences between groups were also investigated in the time-frequency domain using a spectro-temporal cluster permutation test based on the previously identified ROIs. This test was conducted on the same principles as used in the SCPT, with the only change that clusters are built across time and frequency (Maris and Oostenveld, 2007). For this analysis, power spectral density (PSD) was computed following positive and negative feedback was compared between the two groups for each region of interest using spectro-temporal cluster permutation test.

The complex Morlet wavelet transform was applied using a frequency range from 4 to 45 Hz with a spectral resolution of 1 Hz. The number of cycles for each frequency (*f*) was set to *f*/3. The results were z-scored using the mean and standard deviation of the 250 ms preceding feedback. For both types of comparisons (positive feedback and negative feedback), a paired *t*-test was used to identify clusters, with a clustering threshold using a critical alpha level of 0.05, corresponding to a *t*-threshold of 2.0, and a significance level of *p* < 0.05. The number of permutations used was set to 10^4^ permutations.

Similar to the previous section, in order to study how time-frequency clusters affect learning, the “learners” and “non-learners” in our sample were compared on the average activity within each significant cluster (time-frequency cluster of interest; TFOI). This was done by extracting the PSD within the significant time-frequency clusters for each participant and averaging it over time and frequency for each group (i.e., “learners” vs. “non-learners”). This was performed separately for Trauma and Control groups to understand the signal’s contribution to learning accuracy in time and frequency.

## 3. Results

### 3.1. Behavioral analysis

In order to test whether trauma affects feedback-based learning, the two groups (Control vs. Trauma) were compared in terms of their learning accuracy and reaction time. This analysis was done on the complete data set from all subjects. First, MANOVA was used to test the effect of group (Trauma vs. Control) on positive feedback accuracy, negative feedback accuracy, positive feedback reaction time, and negative feedback reaction time. Using Wilks’ lambda, the results showed no significant effect of group on the dependent variables (*F*(4,71) = 0.8567, *p* = 0.4943), leading to the conclusion that there is no difference between the Trauma and Control groups in terms of accuracy or reaction time for positive or negative feedback types. Secondly, to avoid any ceiling effect due to the seven participants who completed less than 480 trials (see Methods section), the analysis was repeated using only the first 160 trials, and the results were found to be similar, with no significant effect for the group on any of the dependent variables (results not shown).

**Figure 3:**
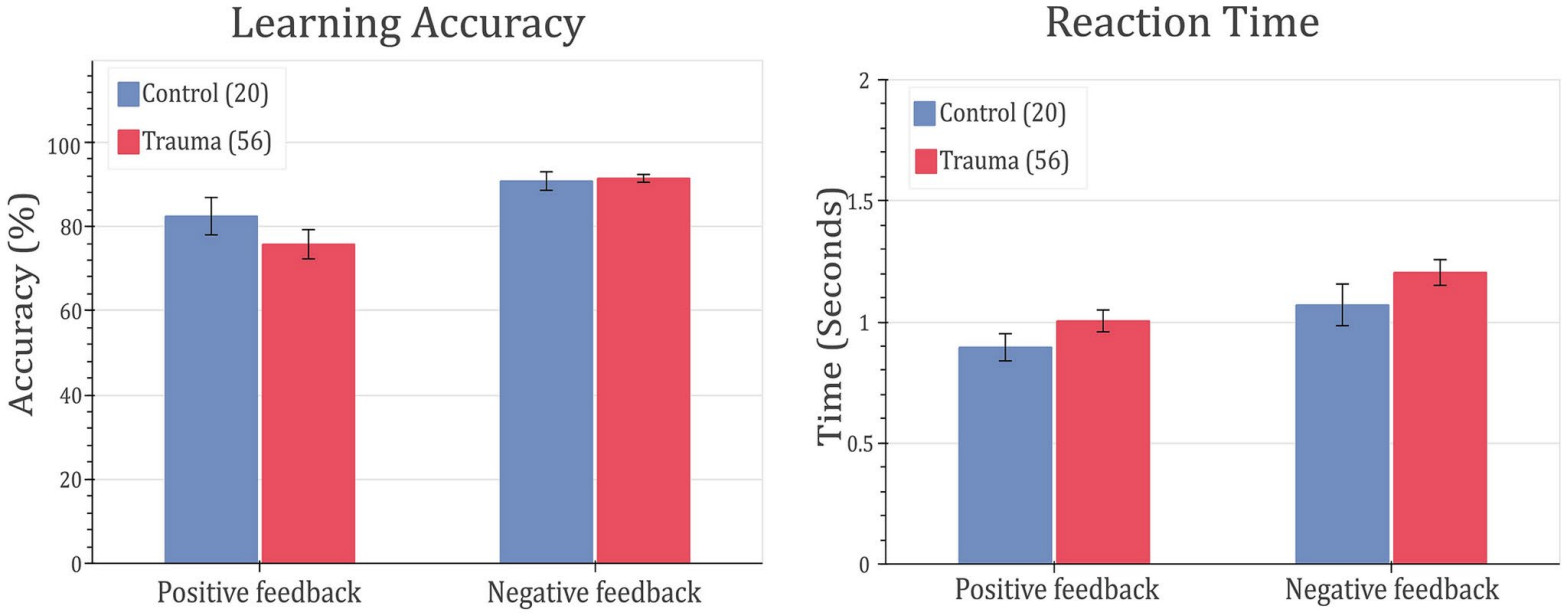
Feedback-learning accuracy and response time. Left: learning accuracy (left) and response time (right) for positive- and negative-feedback trials for Control vs. Trauma groups. Error bars represent the standard error of the mean.

To confirm the findings, the ratio of learners to non-learners was compared in the two groups of comparison. In the tested sample, all participants, except for one, learnt the negative feedback cards more than with more than 65%. Thus, negative feedback is excluded from this analysis. As for positive feedback trials, no significant difference in the learners’ proportions was found between the two groups (*χ*^2^(1) = 0.0019, *p*-value = 0.9651). This further supports the notion that feedback-based learning is not affected in trauma survivors. Behavioral results are shown in Figure 3.

To ensure the absence of confounding effects from other variables in subsequent analyses, the “learners” and “non-learners” groups were compared in terms of gender, age, education level and the 14 modules of the MINI interview. These include major depressive disorder, dysthymia, suicidality, mania, panic attacks, agoraphobia, social phobia, obsessive compulsive disorder, alcoholism, substance use, psychosis, anorexia nervosa, bulimia nervosa, and anxiety. There was no significant difference between the two groups in terms of the proportions of males and females (*χ*^2^ = 0.983, *p*-value = 0.3215), no significant difference in age (learners = 29.23 ± 6.44, non-learners = 32.23 ± 7.89, *t* = −1.6703, *p*-value = 0.0993) and years of education (learners = 17.05 ± 4.02, non-learners = 15.62 ± 4.47, *t* = 1.2895, *p*-value = 0.2018). As for the MINI modules, there were no significant differences between the two groups in any of the modules (smallest *p*-value = 0.2433).

### 3.2. ROI analysis

**Figure 4:**
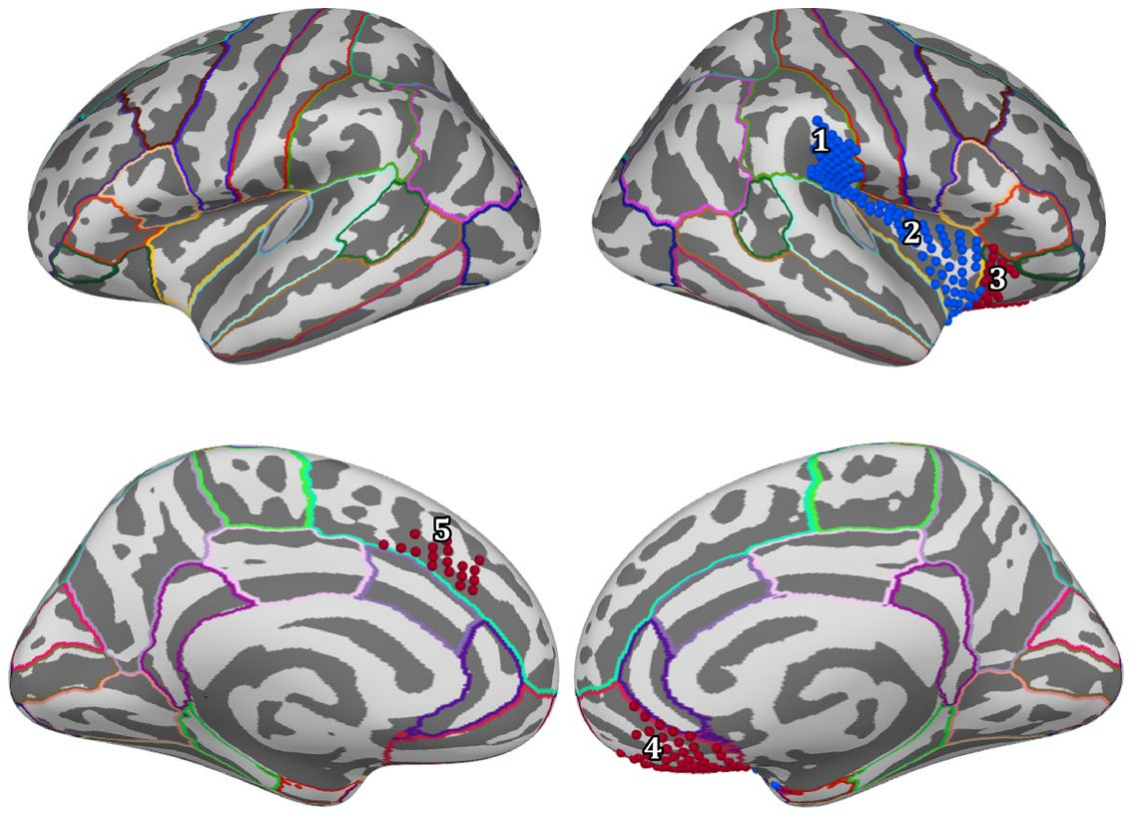
Regions of interest are areas that exhibit a significant difference between the Control group and the Trauma group during positive or negative-feedback trials. Vertices in red indicate higher activity in the Trauma group, while the blue color indicates higher activity in the Control group. Regions #1–4 pertain to positive-feedback clusters, while region #5 pertains to the negative-feedback cluster.

For positive feedback, results from the spatio-temporal cluster permutation test (SCPT) revealed two main clusters that showed significant differences between the two groups in space and time (Figure 4). The first cluster covers the right insula and part of the right supramarginal area as well as a small portion of the lateral part of the lateral orbitofrontal cortex. The activity in this cluster was higher for the Control group (*p*-value = 0.0024). The second cluster covered a large portion of the lateral orbitofrontal cortex as well as the medial orbitofrontal cortex. The activity in this cluster was higher for the Trauma group (*p*-value = 0.0294). By projecting the clusters to brain regions defined by the Desikan-Killiany atlas and eliminating small regions with areas equal to or smaller than 2.29 cm^2^ and regions with short activity duration, four regions were assigned to be the positive-feedback ROIs: supramarginal, lateral orbitofrontal cortex (lOFC) and medial orbitofrontal cortex (mOFC). One cluster was identified in the medial portion of the superior frontal gyrus for negative feedback. This cluster had higher activity in the Trauma group (*p*-value of 0.022). The identified region is considered to be the negative-feedback ROI.

Table 4 details the location of each region and the percentage of the covered area according to the Desikan-Killiany atlas.

**Table 4:** Regions of interest.

#	Region	MNI coordinates			Area (%)	Feedback involvement	Cluster *p*-value
		x	y	z			
1	R-Supramarginal	46	−28	21	26	Positive feedback	0.0024
2	R-Insula	39	0	1	65	Positive feedback	
3	R-Lateral orbitofrontal	28	21	−21	45	Positive feedback	0.0294
4	R-Medial orbitofrontal	6	31	−21	52	Positive feedback	
5	L-Superior frontal	−11	23	35	6	Negative feedback	0.022

The MNI coordinates represent the center of mass for each label. Region (%) refers to the percentage of the active part of the label compared to the entire label.

### 3.3. Temporal dynamics of feedback-related activity

**Figure 5:**
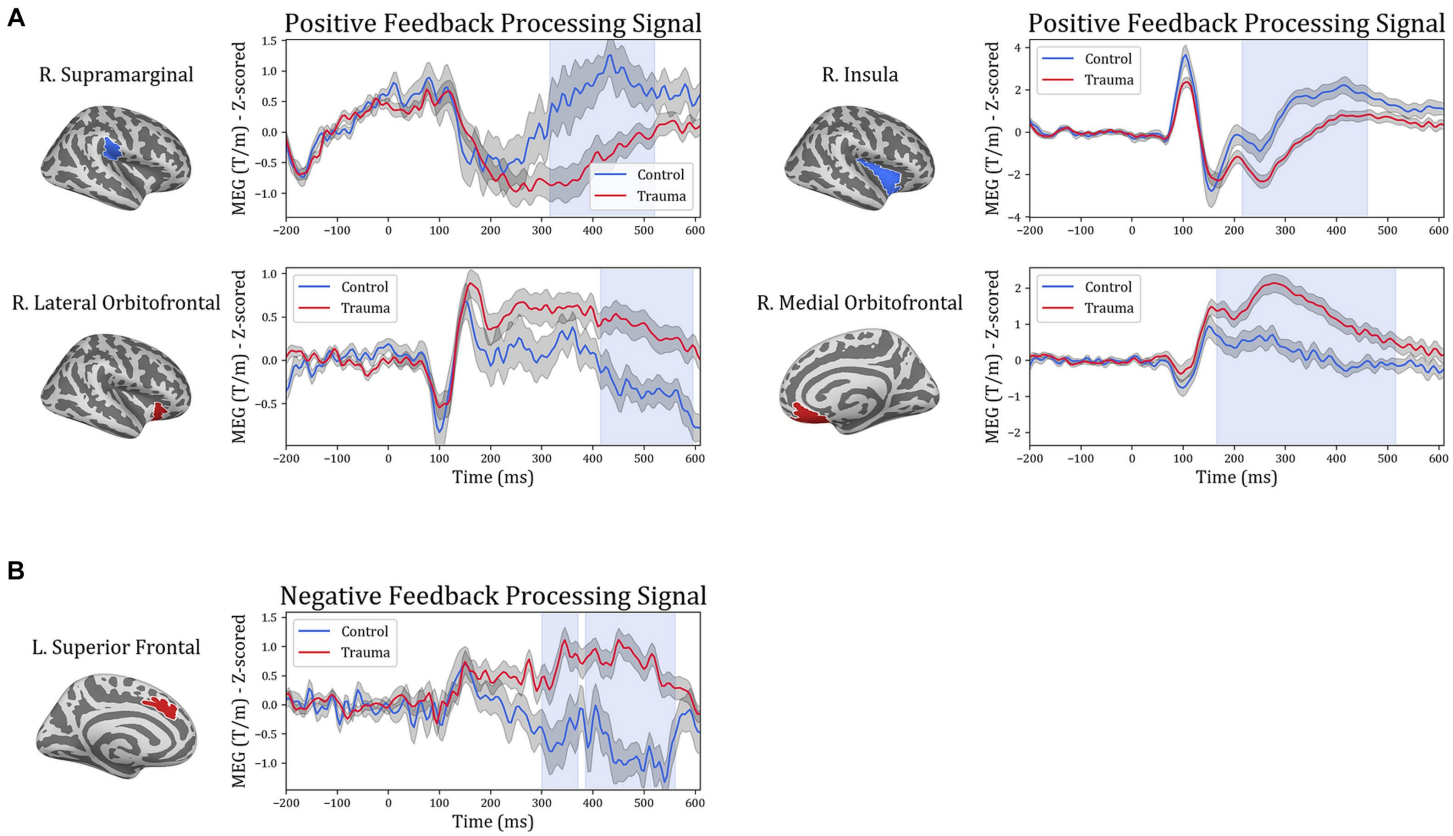
Temporal course of feedback processing of Trauma vs. Control groups. The figure illustrates brain activity in regions that showed significant differences in the time domain for **(A)** positive-feedback trials and **(B)** negative-feedback trials. The light blue shaded area represents the time interval with a significant difference. The gray shaded area around the timeseries represents the standard error of the mean.

In order to gain more insight into the temporal aspect of the differences in signal processing, representative time courses were extracted from the five ROIs and compared between the two groups. The cluster-permutation test in the temporal domain revealed significant clusters in all five ROIs. For positive-feedback trials, the differences span the time interval 160–600 ms after feedback onset. The midway time point for positive-feedback trials was 390 ms (SD = 35 ms). The results of the temporal analysis showed higher activity for the Control group in the insula and the supramarginal cortices, while the Trauma group exhibited higher activity in the lOFC, mOFC, and the medial portion of the superior frontal cortex. This direction of differences is similar to that found using SCPT in the previous section, which confirms the findings reported above. Differences in the temporal dynamics between the two groups and the five brain areas are depicted in Figure 5. The results are also summarized in Table 5.

**Table 5:** Results of temporal analysis.

Region	Cluster periods (ms)	*p*-values
R-Supramarginal	315–525	0.0027
R-Insula	215–465	0.0002
R-Lateral orbitofrontal	415–600	0.0028
R-Medial orbitofrontal	165–520	0.0002
L-Superior Frontal	300–375 & 385–565	0.0090 & 0.0002

The table shows the period of significance and *p*-values for all clusters found.

**Figure 6:**
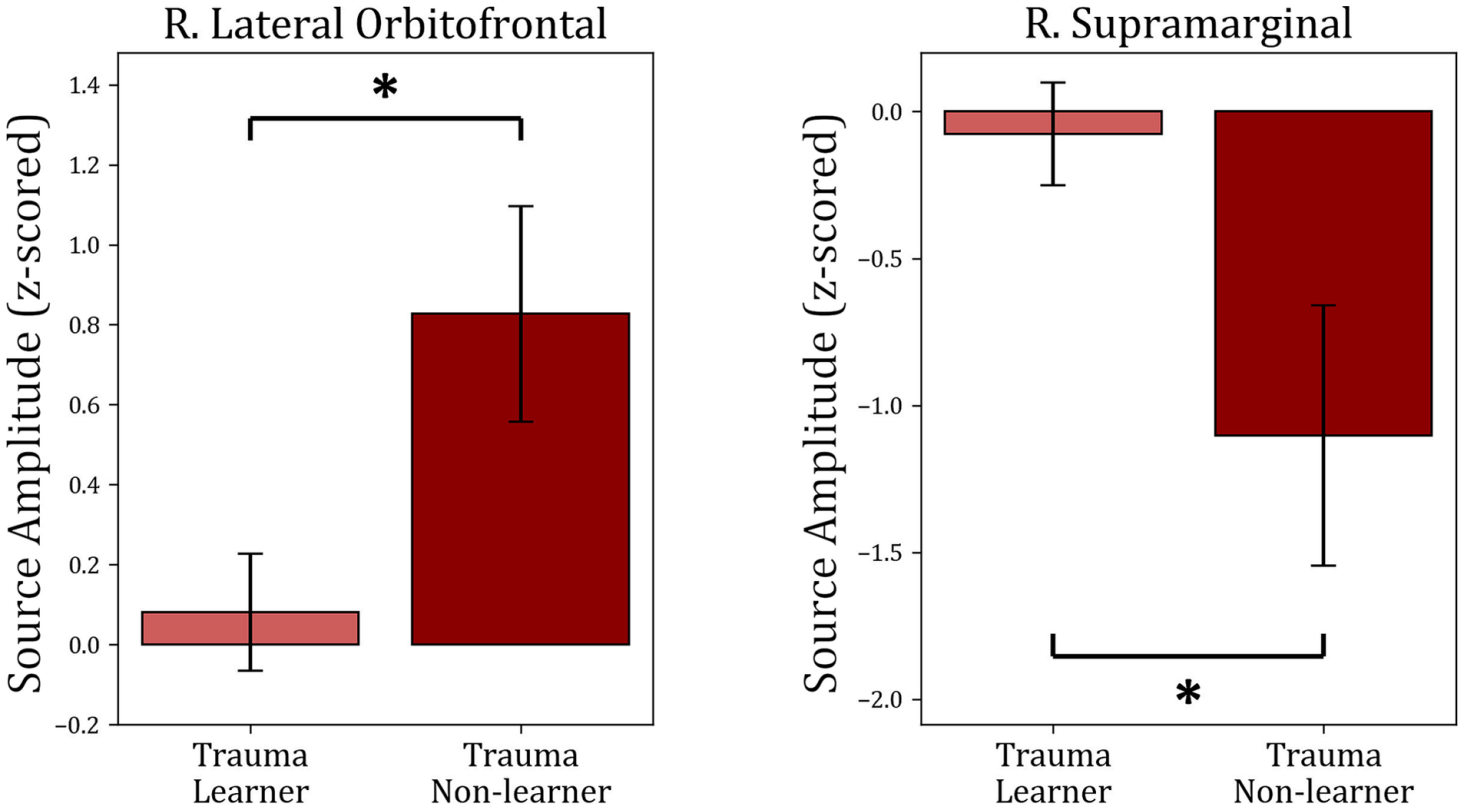
Comparison between “learners” and “non-learners” subgroups of the Trauma group. The figure shows the average source amplitude for the significant time periods found in Figure 5 for both “learners” and “non-learners.” For the supramarginal region, the time period tested was 315–525 ms, and for the lOFC, the period tested was 415–600 ms. The comparison shows significant differences in lOFC and supramarginal cortex. No significant differences were found between “learners” and “non-learners” of the Control group. * *p* < 0.05.

To understand the potential correlations between brain activity within the selected ROIs and learning from positive or negative feedback, the MEG activity within each of the identified time intervals (times of interest; TOIs) was extracted and was used to compare “learners” and “non-learners” in the Trauma and Control groups separately. For the Trauma group, when comparing “learners” and “non-learners,” the results indicate significant differenes in lateral orbitofrontal cortex in the time period 415–600 ms (*t*(53) = −2.664, mean learners = 0.08, mean non-learners = 0.827, *p*-value = 0.01) and the supramarginal cortex in the time period 315–525 ms (*t*(53) = 2.559, mean learners = −0.077, mean non-learners = −1.102, *p*-value = 0.013). Taking the absolute values, we find that the “learners” group have lower activity in both regions compared to the “non-learners” group. No significant differences were found for any of the regions in the Control group (*p*-values >0.18). The results are shown in Figure 6.

### 3.4. Time-frequency analysis

**Figure 7:**
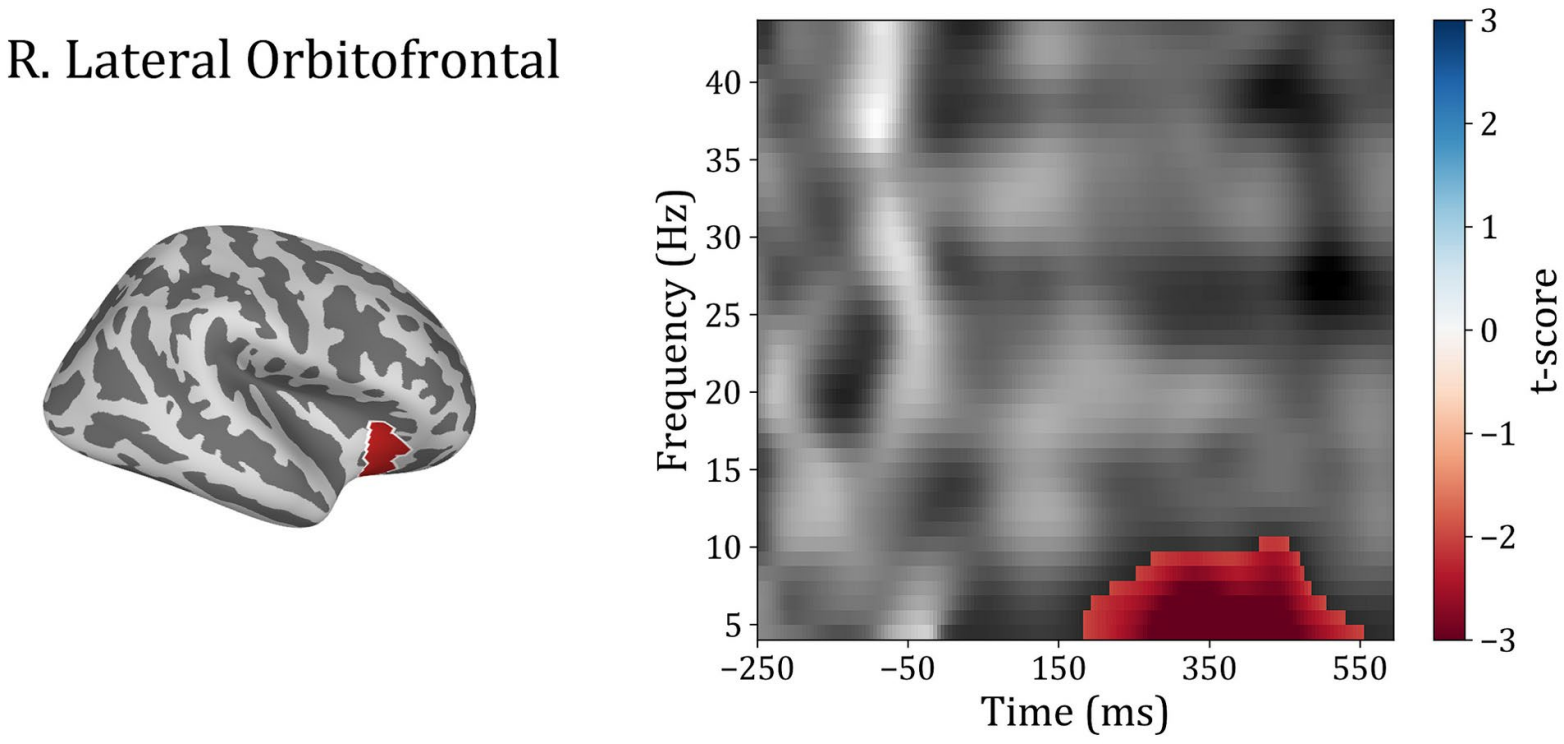
Time-frequency representations of Controls vs. Trauma group comparison. The cluster permutation test revealed a significant cluster (highlighted in red) with higher power in theta (4–8 Hz) and alpha (8–10) bands in the Trauma group. The cluster was found in the right lateral orbitofrontal region in the time window between approximately 185–555 ms where time zero represents the feedback presentation time.

In order to investigate differences between subject groups in the time-frequency domain, the five regions of interest were included to test for differences between Trauma and Control groups for both positive and negative-feedback ROIs. Cluster permutation tests done in the spectro-temporal domain for positive-feedback trials revealed one significant cluster in the lateral temporal lobe that covers the theta band while spanning the interval of 185–555 ms following feedback presentation. Running the analysis on the negative-feedback ROIs did not yield any significant cluster. An illustration of the spectro-temporal test can be found in Figure 7.

**Figure 8:**
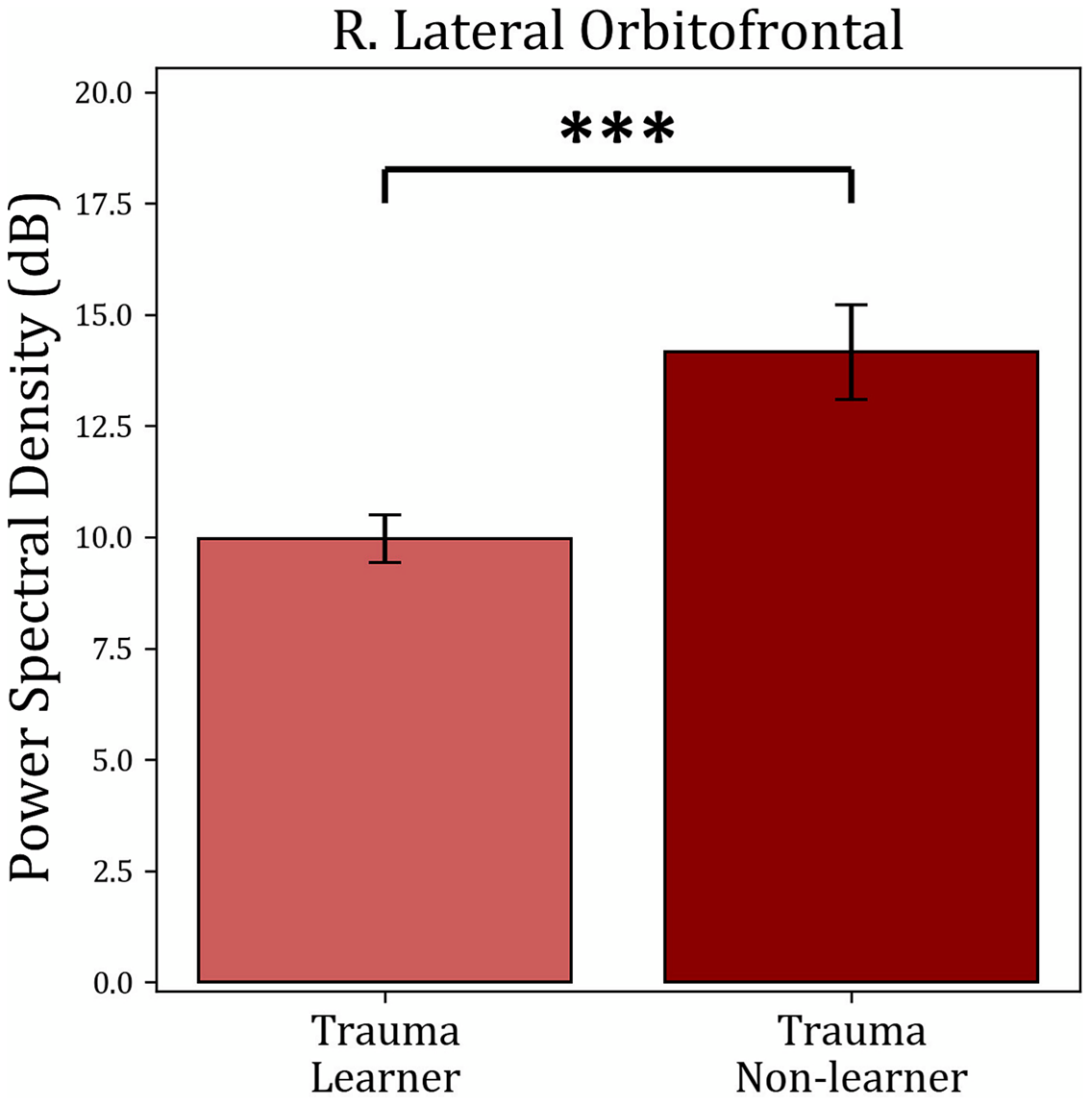
Average power comparison between “learners” and “non-learners” subgroups of the Trauma group. The figure shows the average PSD within the significant time-frequency cluster found in Figure 7 for both the “learners” and “non-learners” groups. The time period tested was 185–555 ms in the frequency range (4–10 Hz). The results show a significantly higher power in the “non-learner” group. No significant differences were found for the healthy controls. *** *p* < 0.001.

The average power within identified TFOIs was compared with positive-feedback scores in for “learners” and “non-learners” in both groups. Trauma group showed a significant difference between “learners” (36) and “non-learners” (19) (*t*(53) = −3.953, mean learners = 9.964, mean non-learners = 14.16, *p*-value <0.001). No significant difference was found in the Control group (*p*-value = 0.353). The results are shown in Figure 8.

## 4. Discussion

The aim of this study was to understand the effect of trauma on the ability to learn from feedback by utilizing MEG to compare brain activity in individuals with a history of trauma with a control group. No significant differences were found in behavioral performance measures, such as accuracy or reaction time, between the two groups. However, by analyzing the brain activity related to feedback processing, significant differences were discovered in the spatial, temporal, and frequency domains. Specifically, when compared to the control group, individuals with a history of trauma displayed increased brain activity in regions such as the mOFC and lOFC and decreased activity in the supramarginal and insular cortices during positive feedback presentation. For negative feedback, the Trauma group exhibited increased activity in the medial part of the superior frontal cortex. These differences occurred relatively late after the presentation of feedback and were characterized by activity within the theta and alpha frequency ranges.

To study the cognitive effects of trauma, learning accuracy was compared between the two groups. The results showed that both groups learned similarly from both positive and negative feedback and exhibited similar reaction times, leading to the conclusion that that response to feedback is not affected in the trauma-exposed group at the crude behavioral level (see Figure 3). Additionally, dividing the sample into learners and non-learners showed no significant difference between the two groups in the positive-feedback trials. On the other hand, almost all participants learnt negative-feedback trials with more than 65% accuracy. This is can be simply attributed to the design of the task, where negative-feedback trials are associated with negative feedback when answered incorrectly, while positive-feedback trials are associated with no feedback. Having no feedback can be also interpreted as absence of negative feedback in our study design, which makes positive-feedback trials more prone to mistakes in learning. This result was the same for both Trauma and Control groups.

These results are consistent with previous studies showing comparable learning and reaction times between trauma-exposed groups and controls (Vythilingam et al., 2009). Other studies have also shown equal learning between a PTSD group and a group of controls (Levy-Gigi et al., 2012; Boukezzi et al., 2020), which suggests that even when the effect of trauma is strong enough to cross the severity threshold for PTSD, it does not seem to affect learning from feedback significantly. Thus, one can conclude that traumatic experiences do not affect the behavioral processing of feedback associated with a stimulus. However, response to feedback may be affected by the trauma at the neural level.

To determine which brain areas respond differently to positive and negative feedback, brain activity was measured in both the Trauma and Control groups during feedback processing using MEG. SCPT was used to compare activity between the two groups while controlling for multiple comparisons. The analysis focused separately on trials when participants received positive or negative feedback. The results showed that there were four regions exhibiting distinct activity between the groups during positive-feedback trials, namely the right supramarginal gyrus, the insula, mOFC, and lOFC. However, during negative-feedback trials, only the medial part of the superior frontal cortex showed a difference in activity.

Previous reports have shown that the aforementioned ROIs are also involved in positive feedback-related processes (see Figure 4 and Table 4). For example, the mOFC and lOFC are involved in forming associations between the stimulus and the feedback (Rolls, 2004) and encoding values to stimuli (Padoa-Schioppa and Assad, 2006; Purves et al., 2018), which is exhibited as higher activity when receiving positive feedback (Sescousse et al., 2013; Oldham et al., 2018; Nguyen et al., 2021). These areas receive input from various sensory regions that provide information about previous experiences to improve value estimation (Radcliffe Hospital et al., 2005; Purves et al., 2018). The insula is more generally activated when positive feedback is received (Wittmann et al., 2010) and is usually involved in the affective processing of positive stimuli and feedback (Sescousse et al., 2013; Nguyen et al., 2021). As part of a larger network, the insula sends information to the OFC to create a representation of the hedonic valence of stimuli (Craig, 2002; Purves et al., 2018; Nguyen et al., 2021). In turn, the supramarginal gyrus connects with the OFC, especially the lOFC (Du et al., 2020), and potentially modulates learning by increasing activity during the retrieval of information acquired through physical enactment (Russ et al., 2003). Accordingly, the supramarginal gyrus may be involved in the process of integrating new information with previous knowledge for future recall. In sum, the processing of positive feedback is a multidimensional process that engages a myriad of neural systems, and our results shed new light on the spatial facets of processing positive feedback with psychological trauma.

Conversely, in terms of the negative-feedback trials, the medial portion of the superior frontal cortex showed increased activity. In general, as a part of the brain’s performance-monitoring system, the superior frontal cortex is activated in response to errors in judgment and helps in avoiding further errors by influencing activity in other relevant brain areas and reducing distracting information (Danielmeier et al., 2011). In essence, one could argue that the increased activity seen in the superior frontal cortex might be the basis of the increased avoidance of negative encounters observed after exposure to trauma. However, in order for this to be proven, an examination of variability in the expression of avoidance symptoms in the Trauma group would be necessary.

The involvement of the insula and orbitofrontal cortices is also supported by the current neuroanatomical theory of PTSD, which indicates that trauma causes hypoactivity in the orbitofrontal cortex and medial prefrontal cortex in response to affective stimuli (Giustino and Maren, 2015; Stark et al., 2015). This could decrease the top-down regulation of amygdala activity and exacerbate symptoms of hyperarousal (Giustino and Maren, 2015; Stark et al., 2015). On the other hand, it has been reported that people who have experienced trauma display an increase in insular activity in response to emotional stimuli (Stein et al., 2007) and that both the insula and medial prefrontal cortex show a decrease in gray matter size following psychological trauma, even in the absence of PTSD (Ganzel et al., 2008).

Interestingly, in the current study, the Trauma group showed decreased activity in the insula but higher activity in the prefrontal, mOFC, and lOFC in response to positive feedback, which may initially seem counterintuitive. However, given that the sample consisted of trauma survivors with no or minimal PTSD symptoms at the time of testing, these results might reflect higher-than-average levels of appraisal of positive stimuli, which might be associated with a better response to trauma. This is further supported by previous studies, which found an increased valuation of positive stimuli in trauma survivors when compared to individuals with PTSD (Myers et al., 2013). This evidence suggests that trauma can cause long-lasting changes to cognition, including more positive feedback reappraisal, which might counter the effects of fear-related and avoidance symptoms. These differences are found at the neural activity level but do not seem to be prominent enough to affect crude measures of task performance at the behavioral level.

In order to further understand the differences in brain dynamics caused by trauma, activity in the regions of interest in the time domain were additionally analyzed (see Figure 5). Changes in processing positive feedback were evident between 165 and 600 ms, which covers two event-related potentials: the p300, and the late positive potential. The p300 is an event-related potential that is seen around 300 ms following feedback reported in EEG studies (Bernat et al., 2015) and usually correlates to secondary aspects of positive feedback, such as those requiring evaluation and comparison (Yeung and Sanfey, 2004; Bernat et al., 2011, 2015). Conversely, the late positive potential starts at 300 ms, which is usually sustained until 2000 ms following positive feedback (Dennis and Hajcak, 2009), is linked to the selective processing of emotional stimuli and activation of emotional systems in response to positive stimuli (Cuthbert and Kozak, 2013). Thus, it is possible to conclude that the differences between the two groups are due to differences in the processing of secondary aspects of positive feedback as well as the emotional processing of positive stimuli. However, since our study design does not directly correlate the processing of secondary emotional aspects or emotional processing with the activity difference between the two groups, we suggest replicating our findings using experiments designed differently before definitively concluding such a correlation.

Analyzing the signal in the frequency domain provided more insight into feedback processing. Feedback processing consists of multiple components, each of which runs in a particular frequency band (Bernat et al., 2015). Using permutation-based clustering tests for the temporo-spectral domain, a cluster of differences was identified in the right lOFC (Figure 7). This cluster of activity spans the time interval of roughly 185–555 ms in the theta frequency band. Although the time interval should not be taken literally due to the nature of the permutation-based clustering test (Sassenhagen and Draschkow, 2019; Meyer et al., 2021), it should at least give an idea about the timing and frequency in which the significant differences were found.

Finally, the activity within each of the identified TOIs and TFOIs was compared between the “learners” and “non-learners” subgroups separately for the Trauma group and the Control group (Figure 6, Figure 8). This comparison was performed to determine whether the difference in learning is reflected in the activity within the TOIs and TFOIs in question. The results show that the activity within the TOIs was different between the two groups in the supramarginal (lower in the non-learners) and the lOFC (higher in the non-learners) regions. Similarly, the average power was significantly higher in the TFOI of the lOFC in the non-learner group. This result can be explained by one of two hypotheses. First, it is possible that the activity within these two regions is correlated with learning from positive feedback. Specifically, the activity in the lOFC region may have a negative impact on learning from positive feedback, while the activity within the supramarginal cortex region may have a positive impact on learning in the trauma group. Meanwhile, this effect is not noticeable on the behavioral level. Secondly, when examining the absolute values, the “learners” group have lower activity in the supramarginal and lOFC TOIs, as well as in the lOFC TFOI. This suggests that they have more attenuated activity in both regions. This can be due to the habituation effect, which refers to a decrease in response to the repeated presentation of a stimulus with emotional valence (Wright et al., 2001). Previous research supports these findings, where repeated presentation of an emotional stimulus resulted in lower brain activity in certain brain regions (Wright et al., 2001). Regardless of the explanation, these results confirm the correlation between brain activity in the lOFC and the supramarginal gyrus with receiving positive feedback. Furthermore, the “non-learners” subgroup seems to be the main contributor to the effects seen between Controls and Trauma in lOFC and supramarginal regions. However, since this learning effect is missing in the “non-learners” subgroup of the Controls, this suggests that the contribution of lOFC and supramarginal regions to learning is potentially perturbed following exposure to psychological trauma.

Taken together, no evidence to show that trauma-exposed individuals differ from individuals with no history of trauma was found at the behavioral level. However, differences between the groups were found at the neural level in the space, time, and frequency domains of cortical activity. These findings provide a deeper understanding of the cognitive processes that are affected as a result of trauma and demonstrate a novel framework for studying the underlying cognitive mechanisms that contribute to psychiatric symptoms by assessing the contribution of different domains of the brain signal on the targeted behavior. To the best of the authors’ knowledge, this is the first study to combine the spatial, temporal, and spectral aspects of feedback processing in individuals with exposure to psychological trauma. It is anticipated that future work will build on these findings to focus on the potential of using cognitive and psychological constructs in assessing symptom improvement following trauma as part of individualized treatment plans.

A limitation of this study is that the impact of trauma was only assessed after exposure, which does not discount the presence of the reported differences before trauma exposure. To address this, future studies should employ a longitudinal design to identify any cognitive and neural differences that develop specifically as a result of exposure to trauma. Additionally, since the ROIs were determined using total signal and there is an inverse correlation between total signal and frequency, it is more likely that our ROIs will exhibit differences in low-frequency bands. This issue can be addressed in the future by selecting an alternative approach to identify the areas of interest.

## Data availability statement

The original contributions presented in the study are included in the article/supplementary materials, further inquiries can be directed to the corresponding author.

## Ethics statement

The studies involving humans were approved by the Ethics Committee at the Medical Faculty of RWTH Aachen, Germany. The studies were conducted in accordance with the local legislation and institutional requirements. The participants provided their written informed consent to participate in this study.

## Author contributions

AS, CK, MH, and JD: conceptualization. AS and FB: data curation. AS: formal analysis, visualization, and writing – original draft preparation. NS and IN: funding acquisition. AS and FB: investigation. NS, IN, MH, and JD: project administration. NS, NK, and IN: resources. AS, CK, FB, MH, and JD: software. IN, MH, and JD: supervision. AS, MH, and JD: validation. MH and JD: writing – review and editing. All authors contributed to the article and approved the submitted version.
